# Anti-Infectives Restore ORKAMBI^®^ Rescue of F508del-CFTR Function in Human Bronchial Epithelial Cells Infected with Clinical Strains of *P. aeruginosa*

**DOI:** 10.3390/biom10020334

**Published:** 2020-02-19

**Authors:** Onofrio Laselva, Tracy A. Stone, Christine E. Bear, Charles M. Deber

**Affiliations:** 1 Division of Molecular Medicine, Research Institute, Hospital for Sick Children, Toronto, ON M5G 0A4, Canada; onofrio.laselva@sickkids.ca (O.L.); tracy.stone@mail.utoronto.ca (T.A.S.); bear@sickkids.ca (C.E.B.); 2 Department of Physiology, University of Toronto, Toronto, ON M5S 1A8, Canada; 3 Department of Biochemistry, University of Toronto, Toronto, ON M5S 1A8, Canada

**Keywords:** CFTR, correctors, antimicrobial peptide, tobramycin, infection, anti-inflammatory, *Pseudomonas aeruginosa*, clinical strains, multidrug resistant bacteria

## Abstract

Chronic infection and inflammation are the primary causes of declining lung function in Cystic Fibrosis (CF) patients. ORKAMBI^®^ (Lumacaftor-Ivacaftor) is an approved combination therapy for Cystic Fibrosis (CF) patients bearing the most common mutation, F508del, in the cystic fibrosis conductance regulator (CFTR) protein. It has been previously shown that ORKAMBI^®^-mediated rescue of CFTR is reduced by a pre-existing *Pseudomonas aeruginosa* infection. Here, we show that the infection of F508del-CFTR human bronchial epithelial (HBE) cells with lab strain and four different clinical strains of *P. aeruginosa,* isolated from the lung sputum of CF patients, decreases CFTR function in a strain-specific manner by 48 to 88%. The treatment of infected cells with antibiotic tobramycin or cationic antimicrobial peptide 6K-F17 was found to decrease clinical strain bacterial growth on HBE cells and restore ORKAMBI^®^-mediated rescue of F508del-CFTR function. Further, 6K-F17 was found to downregulate the expression of pro-inflammatory cytokines, interleukin (IL)-8, IL-6, and tumor necrosis factor-α in infected HBE cells. The results provide strong evidence for a combination therapy approach involving CFTR modulators and anti-infectives (i.e., tobramycin and/or 6K-F17) to improve their overall efficacy in CF patients.

## 1. Introduction

Cystic Fibrosis (CF) is a genetic disease that is caused by mutations in the cystic fibrosis transmembrane conductance regulator (CFTR) gene [1,2,3]. A deficient CFTR function causes the failure of chloride secretion and sodium hyperabsorption at the apical airway surface, leading to the dehydration of the airway surface, impaired mucociliary clearance, and the accumulation of viscous mucus at the epithelial surface [4,5]. As a result, CF patients are prone to contracting bacterial lung infections with the opportunistic pathogen *P. aeruginosa* [6]. Prolonged *P. aeruginosa* infections have been linked to chronic inflammation in the CF lung [7,8], worsening the damage to lung tissue, and leading to eventual respiratory failure [9]. More than 2000 CF-causing mutations have been identified (www.genet.sickkids.on.ca; www.CFTR2.org). The most common mutation, the deletion of phenylalanine at position 508 (F508del-CFTR), induces the misfolding of the protein, causing retention in the ER and degradation by proteasomal pathways [10].

CFTR correctors are pharmacological compounds that rescue the CFTR protein to the cell surface. Lumacaftor (VX-809) has been shown to rescue F508del-CFTR function to approximately 15% of normal channel activity in human bronchial epithelial cells when treated in combination with the potentiator Ivacaftor (VX-770) [11]. The FDA approved the combination Lumacaftor-Ivacaftor (ORKAMBI^®^) for patients bearing the F508del-CFTR mutation. However, clinical studies on ORKAMBI^®^ demonstrated that the response to treatments is variable among patients and the beneficial effects on lung function remain less than expected [12,13]. Low efficacy and high patient-to-patient variation in ORKAMBI^®^ response might feasibly be attributed, in part, to differences in types of microbial infections across patients and even within a single patient over time [14,15]. *P. aeruginosa* (lab strain PAO1) exposure has been shown to reduce corrector-mediated rescue (VX-809 or VRT-325) of CFTR in human bronchial epithelial cells (HBE) [16,17,18] and stimulate the expression of the pro-inflammatory cytokines, IL-6 and IL-8 [18,19]. On the other hand, 4,6,4′-trimethylangelicin (TMA), which is a dual-acting compound (CFTR corrector and potentiator), has been shown to exert its action by interacting directly with the CFTR protein, and reducing *P. aeruginosa*-dependent IL-8 secretion [20,21,22,23,24].

Tobramycin is an FDA-approved aminoglycoside antibiotic that is used for the treatment of *P. aeruginosa* pulmonary infection in CF patients [25]. Tobramycin’s effect on CFTR function remains unclear, despite its demonstrated anti-infective activity. Tobramycin, like Geneticin (G418) and Gentamicin, exhibits read-through ability on premature termination codons (PTCs) mutations [26]. Additionally, cationic antimicrobial peptides (CAPs) represent an important and underutilized resource for combating infection in the CF lung [27,28]. CAPs are generally short, positively-charged, amphipathic peptides that broadly function through the selective disruption of bacterial cell membranes [29,30]. Recently, we showed the ability of the non-amphipathic antimicrobial peptide 6K-F17 to inhibit the growth of clinical multidrug resistant *P. aeruginosa* strains that were isolated from chronically infected CF patients [31]. Further, 6K-F17 is highly effective in disrupting and killing pre-formed multidrug resistant *P. aeruginosa* biofilms [32]. The co-treatment with tobramycin demonstrated that 6K-F17 could potentiate tobramycin bacterial killing activity at low doses by helping to eliminate pre-formed biofilms [32].

Pre-existing infections with clinical strains of *P. aeruginosa* emphasize the importance of coupling ORKAMBI^®^ treatment with anti-infectives to improve the rescue of F508del-CFTR function in vitro. In the current study, we test the ability of tobramycin and 6K-F17 to eradicate lab and clinical strain *P. aeruginosa* infections on top of WT- and F508del-CFTR human bronchial epithelial cells. We show the negative impact that *P. aeruginosa* infections have on WT- and F508del-CFTR function, and how the application of the anti-infectives tobramycin and 6K-F17 reverses infection-mediated decreases in CFTR function.

## 2. Materials and Methods

### 2.1. Human Cell Line

16HBE14o-cells were genome-edited to produce the homozygous CFF-16HBEge CFTR F508del cell line were obtained from the Cystic Fibrosis Foundation [33]. WT and F508del-HBE cells were grown at 37 °C for 5 days post-confluence submerged on 96-well black well, clear bottom culture plates (Costar, Amsterdam, The Netherlands) in Eagle’s minimal essential medium (EMEM) (Wisent BioProducts, Saint-Jean-Baptiste, QC, Canada) with 10% Fetal Bovine Serum (Wisent BioProducts, Quebec, Canada) and 1% Penicillin/Streptomycin (Wisent BioProducts, QC, Canada) [34].

### 2.2. Bacterial Strains

Lab strain *Pseudomonas aeruginosa* (PAO1) was purchased from Dharmacon Inc. (Lafayette, CO, USA) and maintained as a frozen glycerol stock that was stored at −80 °C. Clinical isolates of persister strains of *P. aeruginosa* (214, 287, 330, 380) were obtained with consent from the sputum of cystic fibrosis patients at the Hospital for Sick Children (Toronto, ON, Canada) who had persisting infections post-treatment with inhaled tobramycin [Research Ethics Board (REB) #1000019444]. The cultures were maintained as a frozen glycerol stock that was stored at −80 °C. Consent was obtained from a parent or legal guardian if not of age. All of the methods were performed in accordance with the relevant guidelines and regulations for research involving human subjects at the Hospital for Sick Children. The liquid cultures were generated from the same glycerol stock for each experiment.

### 2.3. Peptide Synthesis and Quantification

6K-F17 (KKKKKK-AAFAAWAAFAA-NH_2_) was synthesized while using standard solid-state Fmoc (Fluorenylmethyloxycarbonyl) [30,31]. (Fmoc-aminomethyl3,5-dimethoxyphenoxy)-valeric acid-polyethylene glycol-polystyrene resin was used to produce an amidated C-terminus. 2-(7-Aza-1H-benzotriazol-1-yl)-1,1,3,3-tetramethyluronium hexafluorophosphate and *N*,*N*-diisopropylethylamine were used as the activation pair. Deprotection and peptide cleavage was performed in a mixture of 88% TFA, 5% phenol, 5% water, and 2% triisopropylsilane for 2 h in the dark at room temperature. Crude peptide was purified on a reverse-phase C4 preparative HPLC column while using a linear gradient of acetonitrile in 0.1% TFA. The peptides were purified to >95%. Pure lyophilized peptide was dissolved in ultrapure water and Trp absorbance at 280 nm recorded. Peptide concentration was determined while using the extinction coefficient 5690 M^−1^·cm^−1^. Peptide stocks were stored at −20 °C and thawed immediately before use.

### 2.4. Infection of HBE Cells

Bacterial overnight cultures were grown from frozen glycerol stocks in tryptic soy broth (TSB) at 37 °C and 250 rpm. 1 mL of overnight culture was used to inoculate 20 mL of fresh TSB and the cultures were grown at 37 °C, 250 rpm to an OD_600_ of 0.5. The cultures were harvested by centrifugation at 4250 rpm for 10 min, and briefly washed with phosphate buffered saline (pH 7.4), before being resuspended in serum and antibiotic free EMEM media to a final OD_600_ of 0.05. A serial two-fold dilution of 6K-F17 in water was prepared in a sterile 96-well microtiter plate, to which infected or non-infected media, with or without VX-809, was added. EMEM media on HBE cells was then replaced with 100 µL of infected or non-infected media containing either peptide, water (positive control for bacterial growth), or tobramycin (10 µg/mL; negative control for bacterial growth). The plates were incubated for four hours at 37 °C with 5% CO_2_ in an incubator designated for infection studies. After four hours, the media was removed from HBE epithelial cells and used in the microbial viability assay to quantify the number of live bacteria. The plates were then either directly frozen (−80 °C) for messenger RNA quantification or incubated with FLIPR dye for CFTR functional studies.

### 2.5. Microbial Cell Viability Assay

Bacteria cell viability was determined while using the BacTitre-Glo^TM^ Microbial Cell Viability Assay (Promega, Fitchburg, WI, USA), as per the manufacturer’s protocol. Briefly, infected media was removed from HBE epithelial cells and incubated with an equivalent amount of equilibrated BacTitre-Glo^TM^ reagent (1:1 ratio) for five minutes in a Greinier opaque luminescence 96-well plate (Sigma-Aldrich, St. Louis, MO, USA). Luminescence was read on a SpectraMax i3X Multi-Mode Assay Microplate reader (Molecular Devices, San Jose, CA, USA). Raw luminescence units (RLU) of non-infected HBE epithelial cells were used to subtract background from infected wells. The background-subtracted luminescence values were normalized to infected HBE epithelial cells incubated with media alone (no peptide or antibiotics) to give normalized bacterial growth (%). Significance was calculated while using one-way ANOVA comparing the mean of infected HBE epithelial cells incubated with media alone to cells incubated with peptide. The incubation with water or media alone was used as a positive control for bacterial growth. Incubation with tobramycin (10 µg/mL) was used as a negative control for bacterial growth. The confirmation of infected and non-infection wells was verified via the microbial viability assay, as well as through streaking on TSB agar plates and incubation for 16 h at 37 °C.

### 2.6. CFTR Channel Function in CFF-16HBEge CFTR Cells

WT and F508del-CFTR cells were grown at 37 °C for five days post-confluence submerged on 96-well black, clear bottom culture plates (Costar) in EMEM media (Wisent BioProducts) with 10% Fetal Bovine Serum (Wisent BioProducts) and 1% Penicillin/Streptomycin (Wisent BioProducts). Twenty-four hours before the assay, the F508del-CFTR HBE cells were treated with DMSO or 3 µM VX-809 in EMEM media with 10% of FBS. The cells were then loaded with blue membrane potential dye dissolved in chloride-free buffer (150 mM NMDG-gluconate, 3 mM potassium gluconate, 10 mM HEPES, pH 7.30, 300 mOsm). The plate was then read in a fluorescence plate reader (SpectraMax i3; Molecular Devices) at 37 °C (excitation: 530 nm; emission: 560 nm). CFTR was stimulated with 10 µM Forskolin (Sigma–Aldrich, St. Louis, MO, USA) and 1 µM VX-770 (Selleck Chemicals) for cells treated with VX-809 for 24 h. The assay was terminated with 10 µM CFTRinh172 (Cystic Fibrosis Foundation Therapeutics). The changes in membrane potential were normalized to the point before addition of agonist and to the DMSO control response [22,35].

### 2.7. RNA Extraction and Quantification (qRT-PCR)

RNA extraction was performed according to the manufacturer’s protocol (Qiagen Micro or Mini Kit) (Hilden, Germany). Briefly, cells were lysed, RNA extracted, and then RNA concentration was measured while using a NanoDrop 2000 instrument (Thermo Fisher Scientific, Waltham, MA, USA). Only samples with a concentration >100 ng/μL were used, with a 260/280 ratio between 1.8 and 2.1. cDNA synthesis was performed while using reverse transcriptase (iSCRIPT cDNA synthesis kit, Biorad, Hercules, CA, USA) or without reverse transcriptase (negative control). Quantitative real-time PCR was performed while using Eva green (Ssofast Evagreen, Biorad, Hercules, CA, USA) fluorophore in 96-well plates (Biorad, Hercules, CA, USA) and then normalized to GADPH. The primers used for amplification are: IL-8 forward: 5′-GACCACACTGCGCCAACA-3′, IL-8 reverse: 5′-GCTCTCTTCCATCAGAAAGTTACATAATTT-3″, TNFα forward: 5′-GGACCTCTCTCTAATCAGCC CTC-3′; TNFα reverse: 5′-TCGAGAAGATGATCTGACTGCC-3′; IL-6 forward: 5′-CGGTACATCCTCGACGGC-3′; IL-6 reverse: 5′-CTTGTTACATGTCTCCTTTCTCAGG-3′; GAPDH forward: 5′-CAAGAGCACAAGAGGAAGAGAG-3′, GADPH reverse: 5′-CTACATGGCAACTGTGAGGAG-3′.

## 3. Results

**Figure 1:**
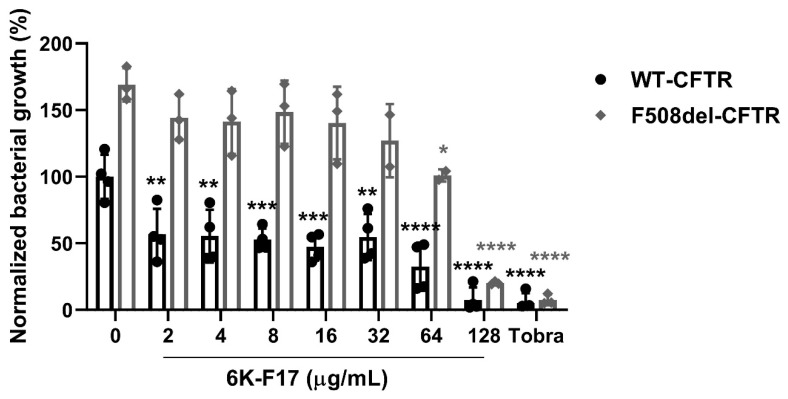
Antimicrobial activity of 6K-F17 on non-Cystic Fibrosis (CF) and CF epithelial cultures. HBE cells were co-cultured with PAO1 bacteria for 4 h with increasing concentrations of 6K-F17 peptide (0–128 µg/mL) or 10 µg/mL tobramycin antibiotic (Tobra). Live bacteria were quantified using the BacTitre-Glo Microbial Cell Viability assay (Promega) and normalized to bacterial growth on WT-human bronchial epithelial cells (WT-HBE) cells alone (0 bar). PAO1 growth on WT-cystic fibrosis transmembrane conductance regulator (WT-CFTR) is depicted by black bars, PAO1 growth on F508del-CFTR in grey bars. Significance is reported as follows: ns, not significant; * *p* < 0.05, ** *p* < 0.01; *** *p* < 0.001; **** *p* < 0.0001. Data shown represent the mean ± SEM (*n* = 3, 4; each dot represents one biological replicate).

Treatment with 6K-F17 decreases bacterial growth on infected WT- and F508del-CFTR human bronchial epithelial cells. We exposed human bronchial epithelial cells (HBE) expressing WT- or F508del-CFTR to laboratory strain *P. aeruginosa* PAO1 bacteria for four hours in the presence of increasing concentrations of antimicrobial peptide 6K-F17 (2–128 μg/mL) and a single dose of tobramycin (10 μg/mL) as a positive control for bacterial growth inhibition. PAO1 bacteria grow more efficiently on F508del-CFTR than WT-CFTR HBE cells, as shown in Figure 1. PAO1 growth decreases in a 6K-F17 concentration-dependent manner for both infected WT- and F508del-CFTR expressing HBE cells. WT-CFTR cells show a significant decrease in bacterial growth at the low dose of 2 µg/mL 6K-F17 (~44% decrease in PAO1 growth), with the nearly complete elimination of bacterial growth at 128 µg/mL (~96%), similar to that observed with (10 µg/mL) tobramycin (Figure 1; black bars). The increased bacterial load on F508del-CFTR HBE cells (70% increase over WT-CFTR cells) requires higher doses of 6K-F17 to lower PAO1 growth (Figure 1; grey bars). In comparison, more than 64 µg/mL of 6K-F17 is required to decrease PAO1 growth by 40% on F508del-CFTR HBE cells. The highest dose of 6K-F17 tested (128 µg/mL) decreased the PAO1 growth by over 80% on F508del-CFTR cells, confirming, for the first time, the ability of 6K-F17 to exhibit antimicrobial activity against *P. aeruginosa* bacteria that were grown atop HBE cells (Figure 1; grey bars) and highlighting the difficulty in eliminating infections on top of F508del-CFTR cells vs. WT-CFTR cells.

6K-F17 and tobramycin restore WT- and F508del-CFTR function in human bronchial epithelial cells that were infected with lab strain *P. aeruginosa*. The FLIPR membrane polarization assay was used to measure forskolin-mediated Cl^−^ secretion to investigate the impact of *P. aeruginosa* infection on WT- and F508del-CFTR function [36]. Forskolin (FSK) was applied to stimulate cAMP-activated Cl^−^ secretion through CFTR, which was later inhibited by the CFTR inhibitor CFTRInh-172. In this manner, we were able to measure the response of CFTR Cl^−^ secretion in HBE cells that were infected with *P. aeruginosa* bacteria in real time in a high through-put manner.

**Figure 2:**
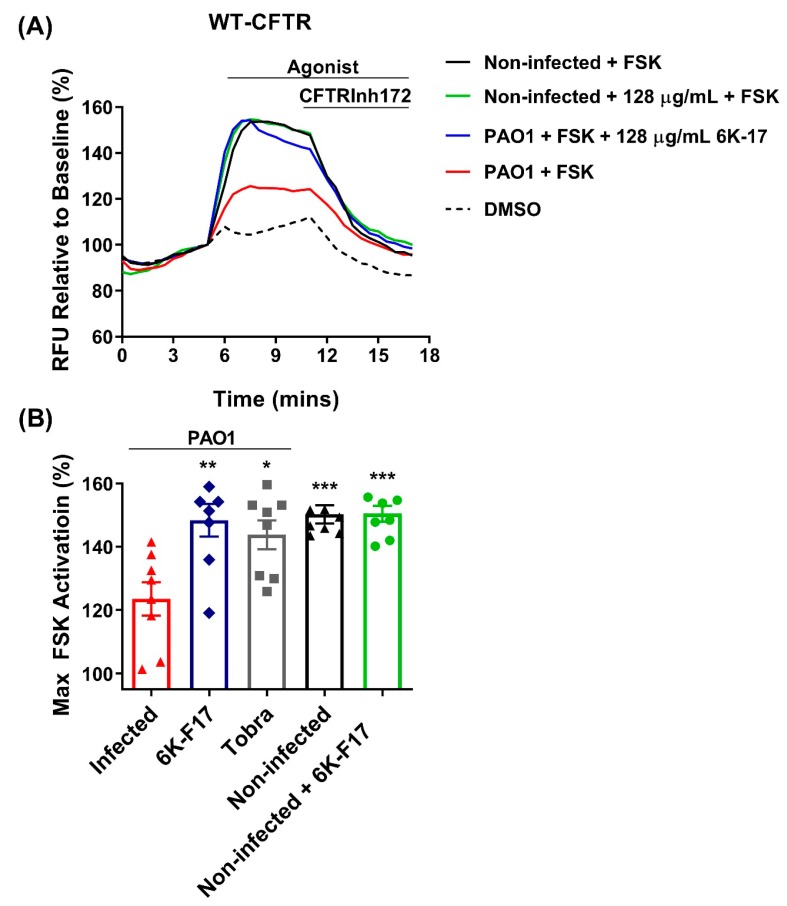
6K-F17 restores WT channel activity in human bronchial epithelial cells infected with *P. aeruginosa*. WT CFTR HBE cells were incubated with 6K-F17 peptide alone (128 µg/mL) or with PAO1 bacteria +/− 6K-F17 peptide (128 µg/mL) or tobramycin (10 µg/mL) for 4 h at 37 °C. (**A**) Representative traces of WT-CFTR dependent chloride efflux while using the membrane depolarization assay. Following a 5 min. baseline measurement, 10 µM Forskolin (FSK) was added. After 10 min, CFTR inhibitor (CFTRinh-172, 10 µM) was added to deactivate CFTR, as noted by the change in the slope of the curves. (**B**) Bar graphs show the mean (±SEM) of maximal activation of CFTR after stimulation by FSK (*n* = 7–8; each dot represents one biological replicate). (* *p* < 0.05, ** *p* < 0.01, *** *p* < 0.001).

**Figure 3:**
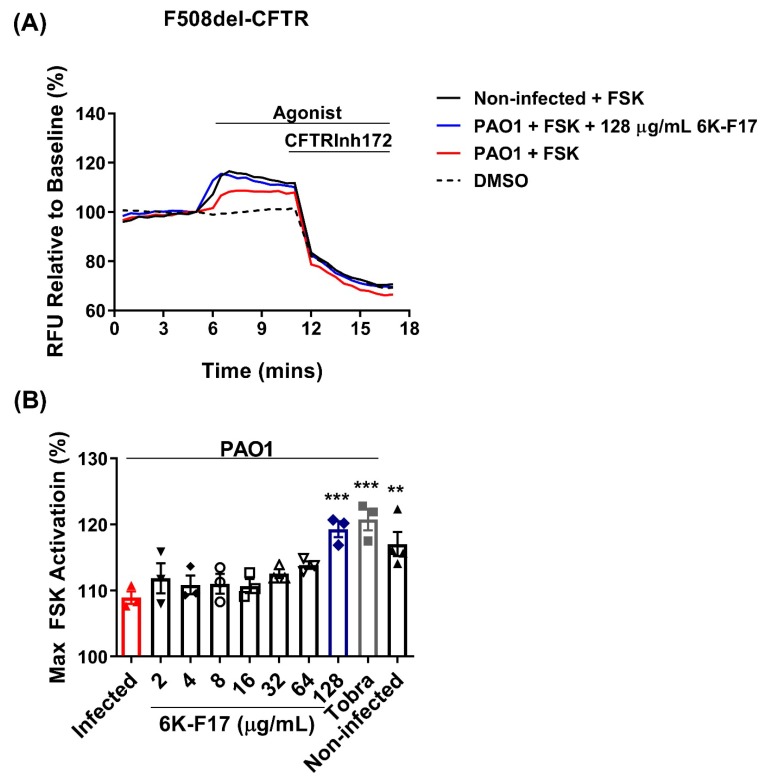
6K-F17 restores F508del-CFTR channel activity in human bronchial epithelial cells infected with *P. aeruginosa*. (**A**) Representative traces of F508del-CFTR-dependent chloride efflux using the membrane depolarization assay. F508del-CFTR HBE cells were incubated with PAO1 +/− 6K-F17 peptide (0–128 µg/mL) or tobramycin (10 µg/mL) for 4 hr. Following a 5 min. baseline measurement, 10 µM FSK + 1 µM VX-770 was added. After 10 min, CFTR inhibitor (CFTRinh-172, 10 µM) was added to deactivate CFTR, as noted by the change in the slope of the curves. (**B**) Bar graphs show the mean (±SEM) of maximal activation of CFTR after stimulation by FSK + 1 µM VX-770 (*n* = 3–4; each dot represents one biological replicate). (** *p* < 0.01, *** *p* < 0.001).

We first investigated the effect of *P. aeruginosa* infection on WT-CFTR function (Figure 2). We found that WT-CFTR function was reduced by approx. 50% after four hours of infection with PAO1 bacteria. Incubation with 128 µg/mL 6K-F17 or 10 µg/mL tobramycin restored WT-CFTR function to that of non-infected cells. We then investigated 6K-F17’s ability to increase mutant F508del-CFTR function in PAO1 infected HBE cells (Figure 3). As previously studied (14–16), we confirmed that infection with PAO1 bacteria decreases F508del-CFTR function (Figure 3). We observe a nearly 50% loss in F508del-CFTR function after four hours of infection. Incubation with 6K-F17 shows a concentration-dependent return of CFTR function to that of non-infected cells (Figure 3B). At a dose of 128 µg/mL 6K-F17 or 10 μg/mL tobramycin, F508del-CFTR function was restored to that of non-infected HBE cells (Figure 3). In comparison to the bacterial growth seen in Figure 1, the restoration of F508del-CFTR function occurs at 6K-F17 concentrations, where there remains up to 20% bacteria growth. The results suggest that 6K-F17 might be able to ameliorate the negative effects of *P. aeruginosa* infection on CFTR function at sub-antimicrobial concentrations.

The infection-induced expression of pro-inflammatory cytokines decreases with the treatment of anti-infective peptide, 6K-F17. *P. aeruginosa* infections in the lung of CF patients have long been associated with an increased inflammatory response and decreased CFTR function [18]. Pathogen-associated molecular pattern molecules (PAMPs), such as bacterial lipopolysaccharides (LPS), have been known to induce pro-inflammatory signaling pathways, including the release of chemokine interleukins [5,7]. The return of CFTR function at concentrations of 6K-F17, where significant bacterial growth is still observed, led us to investigate the expression of the pro-inflammatory cytokines IL-6, IL-8, and TNFα, which have been previously identified to play significant, detrimental roles in CFBE cells when upregulated during infection [8].

**Figure 4:**
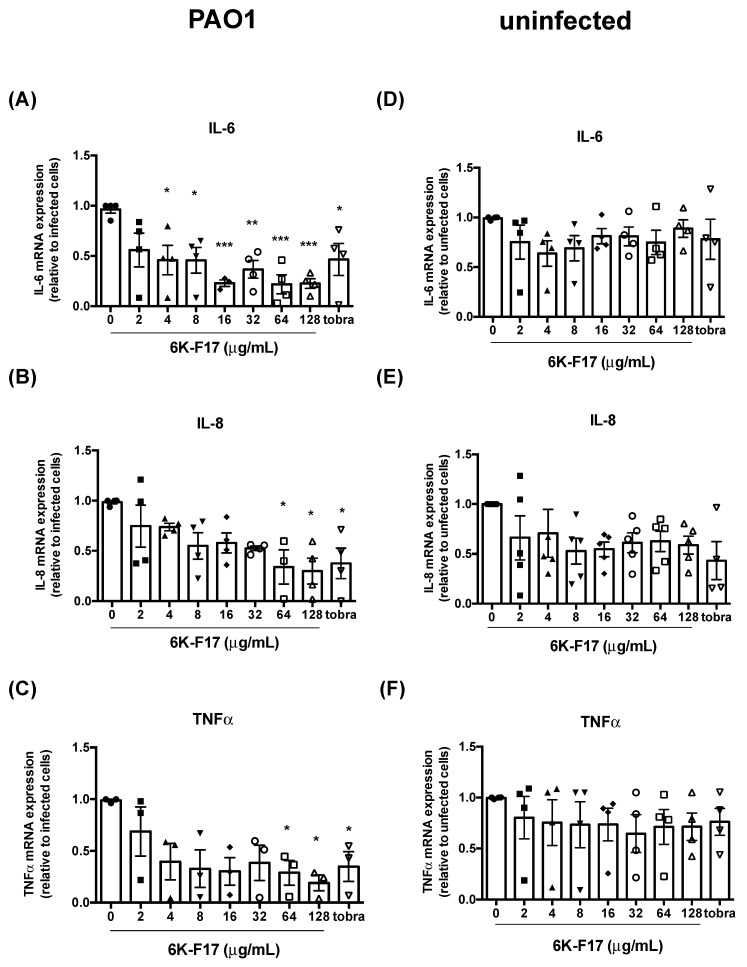
Inflammatory response to PAO1 infection is suppressed by 6K-F17 pre-treatment in human bronchial epithelial cells. (**A**–**C**) F508del-CFTR HBE cells were incubated with PAO1 +/− 6K-F17 or 10 µg/mL Tobramycin (Tobra) for 4 h at 37 °C. (**D**–**F**) F508del-CFTR HBE cells were incubated with 6K-F17 or 10 µg/mL Tobramycin (Tobra) for 4 h at 37 °C. Total RNA was extracted and qRT-PCR was performed in order to quantify IL-6, IL-8 and TNFα mRNAs. Data represent the mean ± SEM (* *p* < 0.05, ** *p* < 0.01, *** *p* < 0.001; *n* ≥ 4; each dot represents one biological replicate).

mRNA expression levels of IL-6, IL-8, and TNFα in PAO1 infected F508del-CFTR HBE cells decrease in a 6K-F17 dose-dependent manner, as shown in Figure 4. A particularly strong response is observed with IL-6, in which a significant decrease is observed at 4 µg/mL 6K-F17 (Figure 4A). The treatment of non-infected F508del-CFTR cells with the same dose of 6K-F7 decreased mRNA expression levels of pro-inflammatory cytokines (Figure 4), however mRNA levels are not statistically different cells not treated with 6K-F17. These data suggest that 6K-F17 in non-infected cells may exhibit some anti-inflammatory activity independent of infection. The incubation with Tobramycin also showed a significant reduction of pro-inflammatory cytokines in F508del-CFTR HBE infected with PAO1, but not in non-infected cells (Figure 4). A comparison to bacterial growth at the same concentrations of 6K-F17 (Figure 1) reveals that significant decreases in pro-inflammatory cytokines are observed when there remains >50% bacterial growth, which suggests that 6K-F17 might be capable of inhibiting activation of inflammatory pathways, likely through the neutralization of pathogen-associated molecular pattern molecules, such as LPS [37].

**Figure 5:**
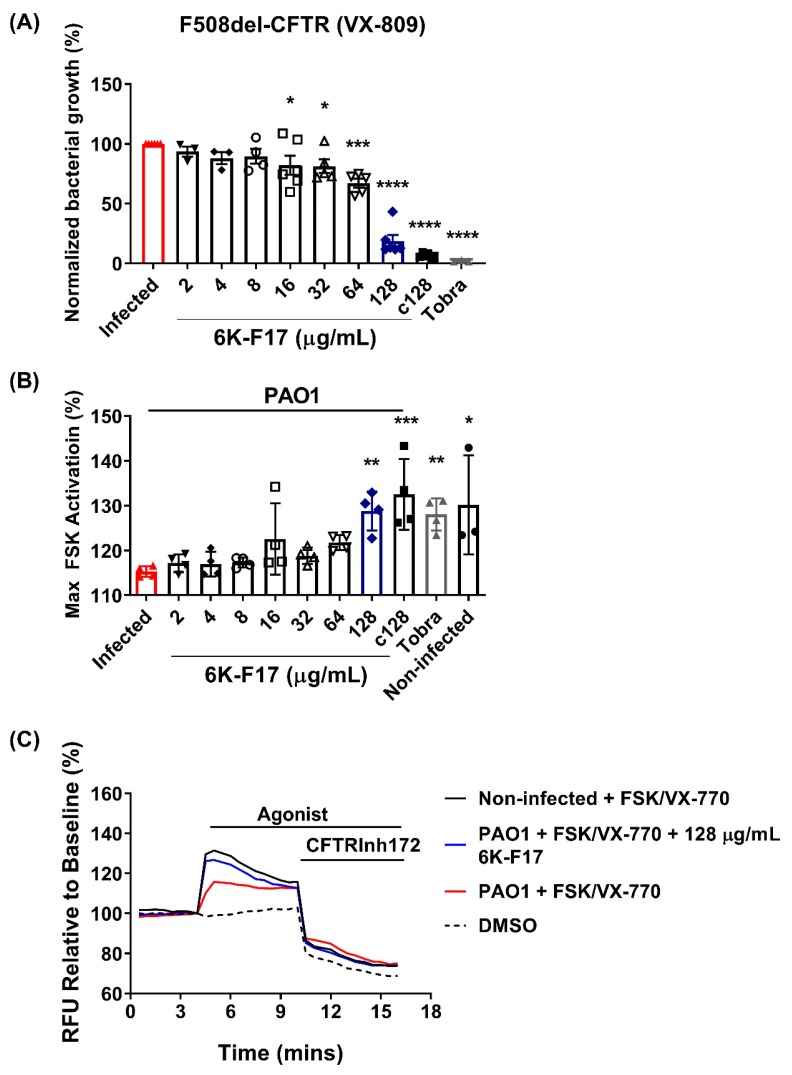
Anti-infectives restore VX-809 rescued F508del-CFTR channel activity in human bronchial epithelial cells infected with *P. aeruginosa*. F508del-CFTR HBE cells were pre-treated with DMSO or 3 µM VX-809 +/− 128 µg/mL 6K-F17 (c128) for 24 h prior to infection with PAO1 +/− 6K-F17 peptide (128 µg/mL) or tobramycin (10 µg/mL) for 4 hr. (**A**) A decrease in PAO1 growth is observed when VX-809-treated F508del-CFTR are also treated with 6K-F17 in a dose-dependent manner. (**B**) Bar graphs show the mean (±SEM) of maximal activation of F508del-CFTR after stimulation by FSK+1 µM VX-770 (*n* = 3–6; each dot represents one biological replicate). (**C**) Representative traces of F508del-CFTR-dependent chloride efflux while using the membrane depolarization assay. (* *p* < 0.05, ** *p* < 0.01; *** *p* < 0.001; **** *p* < 0.0001).

The treatment of F508del-CFTR cells with VX-809 does not decrease infection rate and does not potentiate 6K-F17 anti-infective activity. HBE cells were pre-incubated with VX-809 for 24 h prior to PAO1 infection and treatment with 6K-F17 or tobramycin to assess the impact of the corrector VX-809 on the infection rate and on F508del-CFTR function. The presence of the corrector VX-809 did not lower bacterial load on PAO1 infected F508del-CFTR cells and did not improve the loss of F508del-CFTR function in the infection model (Figure 5). The addition of 6K-F17 results in a dose-dependent decrease in bacterial load, with a significant decrease being observed at the low dose of 16 µg/mL 6K-F17 (~20% decrease in PAO1 growth), and the nearly complete elimination of bacterial growth at 128 µg/mL (~80%)—similar to that observed with 10 µg/mL tobramycin (~95%) (Figure 5A).

Concentrations of 128 µg/mL 6K-F17 and 10 µg/mL tobramycin restore ORKAMBI^®^-mediated rescue of F508del-CFTR function in PAO1 infected cells, returning the F508del-CFTR functional levels to that of ORKAMBI^®^-rescued non-infected cells (Figure 5B,C). Moreover, chronic treatment with 128 µg/mL 6K-F17 (c128) for 24 h prior to infection results in the complete inhibition of bacterial growth and restoration of F508del-CFTR function (Figure 5A,B), which indicates that the peptide is stable on HBE cells over 24 h and it has the potential for preventive care. The results highlight the important role infection plays in the ORKAMBI^®^-mediated rescue F508del-CFTR function.

6K-F17 restores ORKAMBI^®^-mediated rescue of F508del-CFTR channel activity in cells infected with clinical strains of *P. aeruginosa*. Persister strains of *P. aeruginosa* (PA287, PA380, PA214, PA330) isolated from the sputum of CF patients who have previously failed to eradicate infection after having undergone treatment with inhaled tobramycin, were grown on F508del-CFTR HBE cells chronically treated with VX-809. Notably, the clinical strains of *P. aeruginosa* grew less efficiently on F508del-CFTR HBE cells than lab strain PAO1 (Figure S1 in Supplementary Materials)—a feature that likely speaks toward the niche evolution of these strains to grow optimally within patient lungs [14,15]. The decreased efficiency in growth does not translate to these strains being easier to kill; on the contrary, they proved much more difficult to eliminate than PAO1 infections.

**Figure 6:**
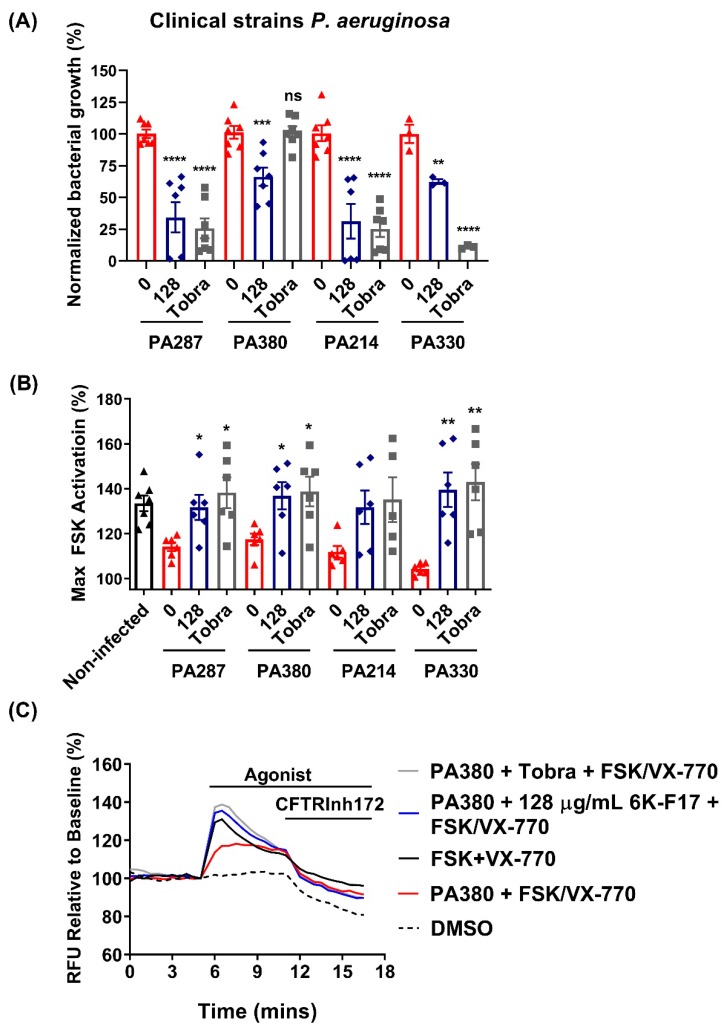
Anti-infectives restore VX-809 rescue of F508del-CFTR channel activity in human bronchial epithelial cells infected with clinical *P. aeruginosa* strains. F508del-CFTR HBE cells were cultured for 24 h with 3 µM VX-809 and co-cultured with clinical *P. aeruginosa* strains isolated from the sputum of CF patients for 4 h +/− 6K-F17 peptide (128 µg/mL) or tobramycin (10 µg/mL). (**A**) Live bacteria were quantified and normalized to bacterial growth on HBE cells alone (0 bar). 6K-F17 significantly inhibits the growth of clinical *P. aeruginosa* strains, in one instance decreasing growth to a tobramycin-resistant strain (PA380) (*n* = 3–6). (**B**) Bar graphs show the mean (±SEM) of maximal activation of CFTR after stimulation by FSK and VX-770 (*n* = 6; each dot represents one biological replicate). (**C**) F508del-CFTR HBE cells were incubated with 3 µM VX-809 for 24 h and co-cultured with clinical *P. aeruginosa* strains +/− 6K-F17 or 10 µg/mL tobramycin antibiotic for four hours. Representative traces of F508del-CFTR-dependent chloride efflux in HBE. * *p* < 0.05, ** *p* < 0.01; *** *p* < 0.001; **** *p* < 0.0001.

6K-F17’s anti-infective activity against the four clinical strains was tested at 128 μg/mL (Figure 6A)—the dose that showed high clearance of bacterial growth and full return of F508del-CFTR function against lab strain PAO1 bacteria. The treatment with tobramycin (10 µg/mL) was included to provide a relative comparison. We found that clinical strains exhibited varying sensitivity to tobramycin and 6K-F17. For two clinical strains—PA287 and PA214—6K-F17 decreased bacterial growth to a similar level to tobramycin (Figure 6A). Extraordinarily, strain PA380, which shows high resistance to tobramycin (100% bacterial growth), is sensitive to 6K-F17 (~40% decrease in bacterial growth). This clinical strain’s response to 6K-F17, but not to tobramycin, represents an excellent example of where traditional antibiotics can fail to eradicate infection, thereby requiring the use of newer non-traditional antimicrobials.

Infection with clinical strains decreased F508del-CFTR function by 48–88%, dependent on the strain (Figure 6B,C). The treatment with 6K-F17 (128 µg/mL) restored ORKAMBI^®^-rescued F508del-CFTR function to that of non-infected cells, including PA330 and PA380, which still exhibited ~60% bacterial growth (Figure 6A). Interestingly, despite the clinical strain PA380 being tobramycin resistant, treatment with tobramycin restored ORKAMBI^®^-rescued F508del-CFTR function. A recent study by Manon et al., demonstrated a correlation between the efficiency of ORKAMBI^®^ to correct CFTR function and the amplitude of decrease in IL-8 mRNA level. These data may suggest that the reduction of pro-inflammatory cytokines by 6K-F17 or Tobramycin restore ORKAMBI^®^-rescued F508del-CFTR function. Full restoration of F508del-CFTR function without a complete decrease in bacterial growth implies that 6K-F17 might exhibit PAMP-neutralizing activity against clinical strains of *P. aeruginosa* as well as lab strain PAO1. Our results also show the beneficial effect of tobramycin treatment on restoring F508del-CFTR function in HBE cells that were infected with both lab strain PAO1 and multidrug resistant clinical strains of *P. aeruginosa* (Figure 6B,C).

## 4. Discussion

**Figure 7:**
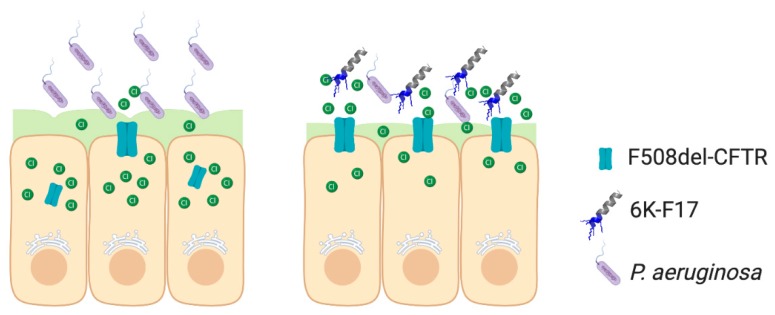
Schematic of *P. aeruginosa* infection of F508del-CFTR HBE cells. HBE cells are depicted in beige, with mucus on top of the apical membrane rendered in green. F508del-CFTR protein is shown in blue with Cl^−^ ions as green circles. *P aeruginosa* bacteria are shown in purple with 6K-F17 peptide (sequence: KKKKKKAAFAAWAAFAA-amide) depicted as grey helices with blue positive charge on one terminus. Bacteria levels and mucus thickness decrease in 6K-F17 treated cells, as apical F508del-CFTR protein levels and Cl^−^ flux increase.

Chronic infection and inflammation are the primary causes of declining lung function in CF patients [6,7,8,9]. Our studies provide the first in vitro evidence that anti-infectives—in addition to eliminating growth of lab and clinical strains of *P. aeruginosa*—help to restore infection-mediated losses in CFTR function (Figure 7). For each cell line tested, infection with lab strain PAO1 bacteria resulted in ~50% decrease in CFTR function relative to non-infected cells (Figure 2, Figure 3 and Figure 5). This broad loss in CFTR function, even in ORKAMBI^®^-rescued F508del-CFTR cells, implies that a similar response to bacteria is responsible for the loss in CFTR function. It has previously been shown that expression of mature CFTR to the apical membrane decreases upon infection with *P. aeruginosa* bacteria [16,17,18], even in cells that were pre-treated with CFTR correctors (VX-809 or VRT-325) [17,18]. Further, Bomberger et al., have shown that outer membrane vesicles secreted from *P. aeruginosa* increase the degradation of apical CFTR by redirecting recycled CFTR from endosomes to lysosomes [38,39]. Thus, it is likely the decreases we observe in WT- and F508del-CFTR function are similarly linked to decreased levels of CFTR at the apical membrane. Infection of ORKAMBI^®^-rescued F508del-CFTR cells with clinical strains of *P. aeruginosa* that were isolated from the sputum of CF patients showed a range in loss of CFTR function, with one strain reducing CFTR activity by as much as 88% (Figure 6C; strain PA330). This observed high variation in F508del-CFTR activity in response to several clinical strains of *P. aeruginosa* might relate to the observed variation in ORKAMBI^®^ response in CF patients in vivo [12,13].

The inflammation in CF has long been recognized to play a detrimental role in CF prognosis, worsening damage to lung tissue, and negatively impacting CFTR function [5,9,40]. We determined the expression levels of mRNA encoding the three pro-inflammatory cytokines IL-6, IL-8, and TNFα to help understand the impact that 6K-F17 treatment has on infection-mediated inflammation. mRNA levels for all three cytokines increased upon infection of F508del-CFTR cells with PAO1 and decreased with subsequent treatment with increasing doses of 6K-F17 (Figure 4). Notably, IL-6 mRNA levels significantly decrease at concentrations of 6K-F17, where ~90% of bacteria are still present (Figure 1 and Figure 4A). The results suggest that 6K-F17 might have protective effects that extend beyond simple antimicrobial activity, preventing the negative impacts of pathogen-induced inflammation at sub-antimicrobial doses. One potential mechanism could be through the neutralization of LPS [41], a PAMP that is known to trigger inflammation signaling pathways [42]. Various other antimicrobial peptides have been shown to interact with and neutralize LPS to lower inflammatory responses to pathogens [19]. The use of 6K-F17 at low—even sub-antimicrobial—dose levels or in combination with a strong antibiotic, such as tobramycin, may therefore prove to be beneficial to minimize detrimental effects of infection-mediated inflammation.

Our approach of using the FLIPR membrane depolarization assay enabled us to record in real time the CFTR-mediated Cl^−^ flux across infected cells in a high throughput manner under several different treatment conditions (e.g., concentration ranges of 6K-F17, with/without VX-809). The coupling of the microbial cell viability assay with the FLIPR membrane depolarization assay provides a direct link between infection status and diminishing CFTR function. This approach could prove valuable for the screening of patient-specific responses to ORKAMBI^®^ or TRIKAFTA rescue under infection conditions, thus personalizing the combination therapy approaches of CFTR modulators with the appropriate anti-infective (i.e., tobramycin and/or 6K-F17) and improving the efficacy in CF patients.

## 5. Conclusions

In the present work, we demonstrate that the antibiotic tobramycin and the cationic antimicrobial peptide 6K-F17 were able to restore the ORKAMBI^®^-mediated rescue of F508del-CFTR function in HBE cells by eliminating the *P. aeruginosa* infection. These data strongly suggest that in vitro screening of patient-specific responses to CFTR modulators under infection conditions could prove to be valuable for personalizing combination therapy approaches with the appropriate anti-infectives to improve the efficacy of CFTR modulators in CF patients.

## Supplementary Materials

The following are available online at https://www.mdpi.com/2218-273X/10/2/334/s1, Figure S1: Normalized *P. aeruginosa* growth in media and atop HBE cells.
