# Supplementary material for: Anti-Infectives Restore ORKAMBI® Rescue of F508del-CFTR Function in Human Bronchial Epithelial Cells Infected with Clinical Strains of P. aeruginosa
Source: Biomolecules. 2020 Feb 19;10(2):334. doi: 10.3390/biom10020334 (PMC7072183; doi:10.3390/biom10020334)
Supplement: Supplementary file 1 [file biomolecules-10-00334-s001.pdf]

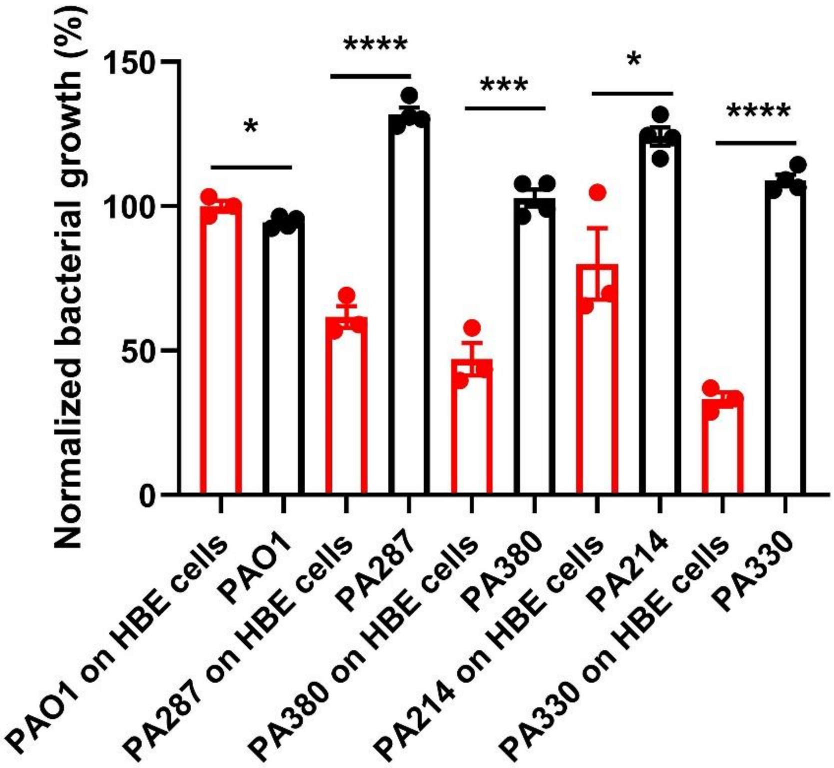

Figure S1.

Figure S1. Normalized *P. aeruginosa* growth in media and atop HBE cells. Background-subtracted luminescence of PAO1 and clinical strains of *P. aeruginosa* grown in cell media (black), or atop of F508del-CFTR HBE cells treated with VX-809 (red). Bacterial load was recorded in the absence of 6K-F17/tobramycin after 4 hr. Significance is reported as follows: ns, not significant; ** $p<0.01$; *** $p<0.001$; **** $p<0.0001$. Data represent mean ± SEM ($n \geq 4$ biological replicates)
